# Sex-Specific Effects of Childhood Poverty on Neurocircuitry of Processing of Emotional Cues: A Neuroimaging Study

**DOI:** 10.3390/bs6040028

**Published:** 2016-12-12

**Authors:** Arash Javanbakht, Pilyoung Kim, James E. Swain, Gary W. Evans, K. Luan Phan, Israel Liberzon

**Affiliations:** 1 Department of Psychiatry and Behavioral Neurosciences, Wayne State University, 3901 Chrysler Service Drive, Detroit, MI 48202, USA; 2 Department of Psychiatry, University of Michigan, 500 S State St., Ann Arbor, MI 48109, USA; jamesswa@med.umich.edu (J.E.S.); liberzon@med.umich.edu (I.L.); 3 Department of Psychology, University of Denver, 2155 South Race Street, Denver, CO 80208, USA; pilyoung.kim@du.edu; 4 Department of Psychiatry, Stony Brook University Medical Center, HSC, T10-020, Stony Brook, NY 11794-8101, USA; 5 Department of Design and Environmental Analysis, Bronfenbrenner Center for Translational Research, Cornell University, Ithaca, NY 14850, USA; gwe1@cornell.edu; 6 Department of Human Development, Bronfenbrenner Center for Translational Research, Cornell University, Ithaca, NY 14850, USA; 7 Departments of Psychiatry, Psychology, Anatomy & Cell Biology, University of Illinois at Chicago, 1747 W. Roosevelt Rd., Chicago, IL 60608, USA; klphan@psych.uic.edu

**Keywords:** poverty, sex, fMRI, amygdala, sex-specific

## Abstract

Background: There is accumulating evidence on the negative impacts of childhood poverty on physical and mental health. Previous work has suggested hyperactive neural response to social fear cues, as well as impairment in neural regulatory functions. However, despite differences found between males and females in stress-related and anxiety disorders, possible sex-specific effects of poverty on emotional processing have not been explored. Methods: We analyzed data from three previously reported experiments of childhood poverty effects on emotional processing and regulation, for sex-specific effects. Participants were 52 healthy Caucasian males and females, from a longitudinal cohort of poverty development study, who were recruited for examining the long-term effects of childhood poverty and stress. The three functional MRI studies included emotion regulation task, emotional face assessment task, and shifted attention emotion appraisal task. Brain activations that associated with childhood poverty previously were entered into a regression analysis with interaction of gender by childhood income-to-need ratio as the independent variable, and age and current income-to-need ratio as variables of no interest, separately for males and females. Results: Amygdala reactivity to implicitly processed fearful faces was positively correlated with childhood income-to-need in adult females but not males. On the other hand, activation in dorsolateral and ventrolateral prefrontal regions during emotion regulation by reappraisal was positively correlated with childhood income-to-need in males. Conclusion: Childhood poverty may exert sex-specific effects in adulthood as presented by hypersensitive emotional reactivity of the amygdala in females, and impaired emotion regulatory function of the prefrontal cortex in males. Results suggest further focus on sex-specific effects of childhood poverty.

## 1. Introduction

Childhood poverty is linked to increased risk of psychopathology and medical illness in adulthood irrespective of adult socioeconomic status [1,2,3,4,5,6]. One in four children in America are born to poverty [7], and identifying underlying mechanisms leading to long-term effects of poverty on physical and mental health is important in developing measures to prevent these adverse effects. There is accumulating evidence for anatomical and functional brain changes in adults as a result of childhood poverty, suggesting a neurobiological nature to the effects of childhood poverty [8,9,10,11,12,13]. Previous work has found this effect in brain regions involved in emotional response and emotion regulation. Changes in amygdala volume, for example, have been repeatedly reported in adults with history of childhood poverty [8,9,10]. Amygdala hyperactivity in response to threat and fear-related social cues has been replicated in emotion provocation studies of adults with history of childhood poverty [11,12,13]. Reduced cortical thickness in anterior cingulate volume was also liked to history of childhood poverty [14,15]. Our group has previously reported that: adults with a history of childhood poverty had lower ventrolateral prefrontal cortex (VLPFC) and (dorsolateral prefrontal cortex) DLPFC activation during cognitive appraisal of their emotional response to emotional faces [16], and in reappraisal of their emotional response to negative pictures [17].

Although evidence for the sex-specific effects of stress and mental illness is abundant and accumulating, the specific neurobiological mechanisms mediating sex-specific effects are largely unknown. Women have a higher chance of developing major depressive disorder [18] as well as other stress and anxiety-related disorders [19]. While affective and anxiety disorders are more common among women, some disorders of regulatory function such as attention deficit hyperactive disorder, conduct disorder [20], antisocial personality disorder [21], and completed suicide [22] are more common in men. Sex-specific differences in stress response might mediate some of the differences noted above. Cortisol is a key hormonal mediator of acute and chronic stress response in humans, and administration of cortisol during fear conditioning reduces activation in emotion regulatory areas (anterior cingulate, medial prefrontal, and orbitofrontal cortex) in men while increasing activation in these areas in females [23]. Cortisol also reduces psychosocial stress-related amygdala responses in men, while increasing it in women [24]. Females in general have larger startle response to threat-related stimuli [25], and larger left amygdala responses to threat-related pictures [26]. Ohrmann and colleagues [27] reported stronger amygdala and prefrontal cortical response to emotional faces in women with panic disorder compared to men with the same disorder. These data suggest a generally larger sensitivity to social cues in women than men [28,29]. There is also evidence for anatomical differences in relation to emotion regulation strategies between men and women. In a study of frontal cortical maturation, Vijayakumar and colleagues [30] found that greater maturational thinning of the DLPFC and VLPFC cortices during adolescence was correlated with cognitive reappraisal abilities in women, but not men.

With regards to sex-specific effects of poverty, we reported that women with childhood poverty had a larger posterior insula response to infant cry than men with history of poverty [12]. There was no main effect of poverty or gender observed in this area, and the observed effect was an interaction of childhood poverty and sex. The effects of childhood poverty in our cohort, however, were not always mediated by the indices of cumulative stress [13,16] suggesting that additional mechanisms might mediate effects of childhood poverty on sex-specific differences. Clearly, more work examining this question is needed, but the existing literature on such sex-specific effects of childhood poverty is very limited. In the current work, we examined data from three (previously reported) emotion provocation [13] and regulation tasks ([12,16] in a cohort of healthy adults with history of childhood poverty, to explore sex-specific effects of childhood poverty in emotional reactivity and regulation. In the context of the previous reports about the sex-specific effects of stress on emotion reactivity and regulation (more pronounced aberrations of emotion reactivity in females and emotion regulation in males), as well as the effects of poverty in the same areas, we expected to see more pronounced amygdala activation in emotion provocation tasks in women with a history of childhood poverty, and less activation in prefrontal cortical areas of DLPFC and VLPFC in emotion regulation tasks in men with childhood poverty. The brain areas which are chosen to explore in this analysis are based on the previous reports of sex differences in emotion processing, the areas that the tasks used in this work are meant to activate, and our previous findings of effects of childhood poverty on brain activation in emotion processing.

## 2. Method

### 2.1. Participants

Healthy unmedicated Caucasian males and females without current or past axis I psychiatric diagnosis confirmed by clinician-conducted Structural Clinical Interview for DSM-IV, enrolled in a 20-year longitudinal cohort of poverty and child development study, were recruited for examining the long-term effects of childhood poverty and stress [13,16,17,31]. The same participants participated in the three tasks reported below. Imaging data for 49 (22 females), 49 (22 females), and 52 (24 females) of these participants were available in the Emotion Regulation Task (ERT) [17], Shifted-Attention Emotion Appraisal Task (SEAT) [16], and Emotional Face Assessment Task (EFAT) [13] respectively for the current analysis. Half of the participants who were recruited at age 9 spent their childhood in low-income households (income-to-need ratio less than 1.5 in New York State) and half grew up in middle-income households (income to need ratio > 1.5). Income-to-need ratio is a per capita index, adjusted annually for costs of living. A ratio equal to or less than 1.0 is defined by the US Census Bureau as “poverty.” All participants were right-handed, and none had a major medical illness or contraindication for MRI (e.g., metallic/ferrous materials in their body). This study was approved by the University of Michigan and Cornell University Institutional Review Boards and all participants provided informed consent. Demographic data are summarized in Table 1. Female and male participants did not significantly differ in age, childhood income-to-need, current income-to-need, or level of education.

### 2.2. Experimental Tasks

Detailed descriptions of the tasks are available in original reports; briefly, in the EFAT task [13] designed to examine amygdala reactivity, participants were asked to match one of the two faces on the bottom to the emotion expressed by target face on top. These included angry, fearful, happy, and neutral faces. In participants with history of childhood poverty, we have observed larger amygdala response to Fearful > Happy faces, due to larger response to fearful, and smaller response to happy faces. In other words, the lower the childhood income-to-need, the higher the response in amygdala to fearful faces, and the lower to happy faces. In the ERT task [17], participants were instructed to “Look” at the neutral IAPS pictures presented on the screen, “Maintain” (attend and experience naturally) the emotional state elicited by the aversive pictures (probing explicit emotional response), or “Reappraise” and voluntarily decrease the level of their negative affect in response to aversive pictures through cognitive reappraisal [32]. Here we observed diminished emotion regulatory activation during reappraisal in the DLPFC and VLPFC in relation to poverty. In the SEAT task [16], participants saw a picture of a fearful or neutral face superimposed on a place, and were asked to either identify the gender of the face (Male/Female), whether the place is indoor or outdoor (In/Out), or if they liked or disliked the face (Like/Dislike). Childhood poverty was correlated with impaired DLPFC function during appraisal-related emotion regulation (Like/Dislike > Male/Female) for this task.

In summary, the EFAT examines implicit emotional reactivity of the amygdala to negative and positive emotional faces, SEAT examines implicit regulation of emotional reactivity by appraisal or attention, and ERT examines explicit regulation of emotional response by reappraisal.

#### 2.2.1. Acquisition of MRI Data

All scanning was performed using a Philips 3 Tesla MRI scanner (Philips Medical Systems, Andover, Massachusetts) in the functional MRI laboratory at the Veterans Affair Ann Arbor. A total of 240 T2*-weighted echo planar gradient-recall echo volumes (echo time = 30 ms, repetition time = 2000 ms, 64 × 64 matrix, flip angle = 90 degree, field of view = 22 cm, 42 contiguous 3 mm axial slices per volume), were acquired for each task. Five additional volumes were discarded at the beginning of each run to allow for equilibration of the MRI signal. A high-resolution T1-weighted structural image was also obtained to provide for more precise anatomical localization.

#### 2.2.2. MRI Data Analysis

Details of the preprocessing and first-level analysis can be found in the original reports describing data analysis for each of the tasks [13,16,17]. Data were analyzed using the statistical parametric mapping software package, SPM8 (Welcome Department of Cognitive Neurology, London, UK).

For the EFAT task, first-level models consisted of regressors for task conditions (angry, fearful, happy, neutral blocks) as well as nuisance regressors consisting of the motion correction parameters from the realignment preprocessing step. We extracted betas for Fearful > Happy contrast using bilateral anatomical (AAL) amygdala masks to examine possible sex-specific implicit emotional response to the faces. We then entered these extracted betas into a regression analysis with childhood income-to-need ratio, gender, and their interaction as the independent variable, and age and current income-to-need ratio as variables of no interest.

For the ERT task, the first-level contrasts included Look (baseline response to neutral images), Maintain > Look (Explicit emotional reactivity), and Reappraise > Maintain (reappraisal-related emotion regulation). A multiple regression was performed with the brain activation as dependent variable, the childhood income-to-needs ratio as an independent variable and the current income-to-needs ratio and age as a covariate of no interest. An initial voxel-wise threshold of *p* < 0.005 and a minimum cluster size of 265 voxels for the Reappraisal vs. Maintain contrast gave a corrected *p* < 0.05. The Region of Interest (ROI) for VLPFC was created by placing an 8 mm radius sphere at the left VLPFC peak (*x*, *y*, *z* = −50, 22, 6). To examine reappraisal-related emotion regulation, betas from this ROI, and DLPFC region (*x*, *y*, *z* = −40, 12, 28; 343 voxels; *p* < 0.05, corrected) were extracted for the contrast Reappraise > Maintain. To ascertain effects of explicit emotional response and explore their consistency with the effects found to the implicit emotional response during EFAT, the “Maintain > baseline” contrast in the ERT task was created to probe explicit emotional reactivity in amygdala. We thus extracted betas for the contrast “Maintain > baseline” for the left and right amygdalas using anatomical AAL bilateral amygdala masks. We then entered these extracted betas into a regression analysis with childhood income-to-need ratio, gender, and their interaction as the independent variable, and age and current income-to-need ratio as variables of no interest.

For the SEAT task, first-level contrast was Like/Dislike > Male/Female (which probes appraisal-related emotion regulation). To ascertain presence of sex-specific effects on implicit emotion regulation, and explore their consistency with the effects found during explicit emotional regulation in the ERT, betas for the DLPFC region were extracted from Like/Dislike > Male/Female contrast using an AAL anatomical mask. We then entered these extracted betas into a regression analysis with childhood income-to-need ratio, gender, and their interaction as the independent variable, and age and current income-to-need ratio as variables of no interest.

Analyses were done using the SPSS software (version 21, IBM, Armonk, NY, USA), with a *p* value threshold set at <0.05.

## 3. Results

Results of the regression analysis are summarized in Table 2. For brevity, only areas where there is an effect of gender, or gender by childhood income-to-need ratio are presented in the table.

### 3.1. Emotional Face Assessment Task (EFAT)

**Figure 1:**
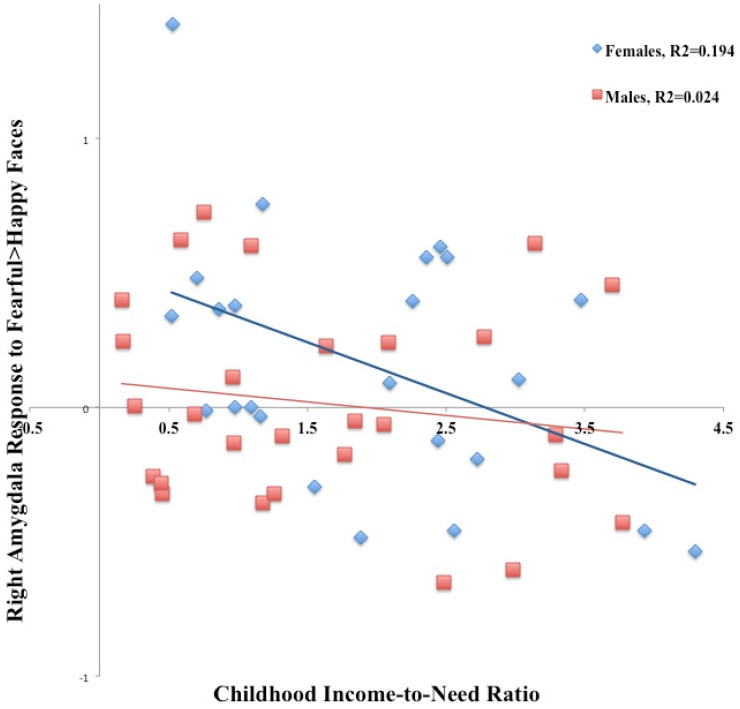
Right amygdala activation in response to Fearful > Happy faces in males and females in the Emotional Face Assessment Task (EFAT) tasks.

Childhood income-to-need ratio (*B* = −0.78, *t*(51) = −2.86, *p* = 0.007), but not gender (*B* = −5.43, *t*(51) = −1.75, *p* = 0.087) predicted brain activation in right amygdala in the contrast Fearful > Happy. There was a significant interaction of gender and childhood income-to-need ratio predicting brain activation in right amygdala in this contrast (*B* = 0.80, *t*(51) = 2.25, *p* = 0.03). Betas from regression were significantly different between males and females (*t*(48) = 5.10, *p* < 0.001), Figure 1. To determine the direction of changes contributing to the observed effect, we did the same regression analysis for Fearful, and Happy faces separately, in both males and females. Right amygdala activation in response to Fearful faces was predicted by childhood income-to-need ratio (*B* = −0.65, *t*(51) = −2.42, *p* = 0.02), gender (*B* = −10.80, *t*(51) = −3.58, *p* = 0.001), and interaction of gender and childhood income-to-need ratio (*B* = 0.86, *t*(51) = 2.48, *p* = 0.017). Betas were significantly different between males and females (*t*(48) = 10.56, *p* < 0.001). Right amygdala activation in response to Happy faces was not predicted by interaction of gender and childhood income-to-need ratio (*B* = −0.36, *t*(51) = −0.97, *p* = 0.34).

Left amygdala activation in the contrast Fearful > Happy was predicted by childhood income-to-need ratio (*B* = −0.70, *t*(51) = −2.47, *p* = 0.02), but not by gender (*B* = −3.75, *t*(51) = −1.16, *p* = 0.3). Gender interaction by childhood income-to-need ratio did not predict brain activation in left amygdala in the contrast Fearful > Happy (*B* = 0.57, *t*(51) = 1.53, *p* = 0.13).

### 3.2. Emotion Regulation Task (ERT)

To examine sex-specific explicit emotional reactivity in the amygdala in response to negative IAPS pictures, we examined the contrast “Maintain > baseline”. In this contrast, childhood income-to-need ratio, gender, or the interaction of gender and childhood income-to-need ratio did not predict activation in left (*B* = 0.60, *t*(48) = 1.57, *p* = 0.13) or right amygdala (*B* = −0.32, *t*(48) = −0.72, *p* = 0.48).

To assess reappraisal-related emotion regulation in the left DLPFC and VLPFC in association with childhood poverty for males and females, separately, we examined the contrast Reappraise > Maintain (reappraisal-related emotion regulation). Left DLPFC activation was predicted by childhood income-to-need ratio (*B* = −5.41, *t*(51) = −1.20, *p* = 0.05), gender (*B* = −5.01, *t*(51) = −2.24, *p* = 0.03), and interaction of gender and childhood income-to-need ratio (*B* = 5.71, *t*(48) = 2.18, *p* = 0.03). Betas were significantly different between males and females *t*(45) = 3.99, *p* < 0.001). In the same contrast, left VLPFC activation was marginally predicted by childhood income-to-need ratio (*B* = −5.67, *t*(51) = −1.84, *p* = 0.07), and significantly predicted by gender (*B* = −5.20, *t*(51) = −2.05, *p* = 0.046) and interaction of gender and childhood income-to-need ratio (*B* = 5.78, *t*(48) = 1.95, *p* = 0.05). Betas were significantly different between males and females *t*(45 = 2.54, *p* = 0.014).

### 3.3. Shifted-Attention Emotion Appraisal Task (SEAT)

To examine sex-specific differences in implicit emotion regulation using appraisal, we examined the contrast Like/Dislike > Male/Female. In the contrast Like/Dislike > Male/Female (appraisal-related emotion regulation), left DLPFC activation was marginally predicted by childhood income-to-need ratio (*B* = 0.49, *t*(51) = 1.81, *p* = 0.07), but not predicted by gender (*B* = 1.34, *t*(51) = 0.43, *p* = 0.7) or interaction of gender and childhood income-to-need ratio (*B* = −0.45, *t*(48) = −0.13, *p* = 0.9).

## 4. Discussion

In this work, we aimed to explore sex-specific effects in a sample of healthy adults with history of childhood poverty. We examined sex-specific effects in implicit and explicit emotional reactivity, using EFAT and ERT accordingly. We also examined sex-specific effects of childhood poverty on implicit and explicit emotional regulation, using appraisal condition in SEAT and reappraisal condition in ERT, accordingly. Our findings suggest that the effects of poverty on implicit emotional reactivity in amygdala are mainly seen in females. The previously observed negative bias toward social fear cues (fearful faces) and away from positive cues (happy faces) in the EFAT task was derived by the female group. This effect was specifically more pronounced on the right side. Interestingly, this effect was not present during explicit emotional reactivity (ERT Maintain > baseline contrast). On the other hand, deficits in brain activation in explicit reappraisal-related emotion regulation were an effect of male participants.

Although human studies of sex-specific effects of early life stress on brain anatomy and function are scarce, animal research provides intriguing evidence for sex-specific effects. Hypothalamus Pituitary Axis (HPA) reactivity is more susceptible to prenatal stress in female rats such that adult female rats show larger corticosterone level at baseline and in response to stress [33,34]. Female rats with early life stress exhibit greater nociceptive responses than males with early life stress [35,36]. Even central administration of female hormones can induce visceral hypersensitivity in female rats [37]. In humans, the social stress test, which triggers a robust HPA response and increases cortisol level, shows sex-specific effects on fear conditioning. This effect is very much in concert with our findings and suggests reduced prefrontal activation in males, and increased amygdala activation in females in response to the fear-conditioned stimulus [24]. Also, administration of cortisol in a fear-conditioning study led to reduced activation in prefrontal regulatory areas in men, and increased activation in these areas in women in response to conditioned stimulus [23]. Our findings suggest sensitized implicit amygdala reactivity to emotional faces as an effect of childhood poverty in adult females. This is in line with previous reports of larger amygdala response to threat pictures in healthy females [26], and emotional faces in female patients with anxiety disorders ([27]. Indeed, prevalence of anxiety disorders is higher in adolescents with history of childhood poverty (for a review see [5]). It is thus possible that a more sensitive salience detection in female amygdala in response to social cues [28] may lead to sensitization to negative social signals as an effect of early life stress, as well as to subsequent development of anxiety disorders with heightened threat detection. Explicit awareness of the emotional response to aversive stimuli (ERT Maintain task) did not show the sex-specific effects of poverty in amygdala activity, which were observed during implicit emotional reactivity (EFAT task). Emotional faces processed implicitly might be a more sensitive probe of amygdala reactivity than explicit processing of emotional pictures that might involve some measure of cognitive processing. Interaction effects in amygdala were lateralized, and seen on the right side and in females. Multiple studies have previously shown left amygdala response to emotional pictures, or recall of emotional memories in females, and right amygdala response in males [26,38,39]. Absence of similar findings in the left amygdala in our study (*B* = 0.565, *t*(48) = 1.53, *p* = 0.13) could be due to the small sample size and low power. On the other hand, right amygdala response could be more vulnerable to the effects of poverty.

Our findings of impaired function in the emotion regulatory areas as an effect of poverty in males is consistent with some earlier findings reported in the animal and human literature. Several animal studies have shown that adult male, but not female, rats with prenatal or infancy exposure to stress had decreased dendritic complexity in medial prefrontal cortex (mPFC) and prelimbic cortex [40]. Blaze and colleagues [41] showed sex-specific changes in methylation of *BDNF* and *reelin* genes in the mPFC in male mice maltreated during infancy. Their findings suggest sex-specific epigenetic changes in expression of genes with a role in brain development and synaptic plasticity in emotion regulatory areas in male mice. Similarly in human studies, [27] cortisol reduced mPFC activation in fear-conditioning paradigm in males, but not females. This indirect evidence is consistent with our findings, and together suggest that structure and function of the prefrontal emotion regulatory areas in males might be more sensitive to developmental effects in general and to stress effects in particular.

Several limitations of the reported findings have to be acknowledged. Our low sample size might have led to limited power in detection of gender by childhood income-to-need interaction. The imaging tasks were not designed to probe differences in emotional processing between males and females, thus more gender-specific paradigms and analyses (e.g., differential response to female or male threat faces, or gender-specific negative images) could provide more specific data for possible threat-specific differences in neural responses of men and women. Secondly, while the absence of sex-specific effects in amygdala reactivity in the ERT task and the presence of these effects in the EFAT could be, as we suggested, due to difference between explicit and implicit emotional reactivity, other between-task differences could have contributed to these “inconsistencies.” ERT tasks involved presentation of aversive IAPS images, and not faces, and some previous studies reported higher amygdala activation in response to negative emotional faces than to negative IAPS images [42,43]. Weaker responses to pictures compared to faces may have reduced our power of detecting possible effects in amygdala in response to IAPS pictures. Furthermore, as is inherent to the majority of laboratory studies, complexity and intensity of the emotional stimuli are lower than in real-life experiences. Hence, expanding the results into in vivo emotion processing should be done cautiously. Finally, our work does not address the question of which mechanisms accompanying early life experience of poverty can lead to differential effects in neurocircuitry of emotion processing between males and females.

## 5. Conclusions

In summary, this is the first report on sex-specific effects of childhood poverty on social emotional processing and regulation. Our findings suggest differential effects of childhood poverty, such that in females it is associated with higher threat detection response in amygdala (and smaller response to positive social cues), while in males with impaired function of emotion regulatory regions. Our findings underline the importance of exploration of possible differential effects of early life stress, as well as poverty in larger studies, as these differences have been rarely explored. These results also suggest exploring possible sex-specific behavioral consequences of childhood stress (e.g., possibility of higher avoidance behavior due to higher threat detection in females versus impulsivity in males). However, at this stage, more studies exploring these differential effects are needed to map the potential differences in underlying neurocircuitry.

## Figures and Tables

**Table 1:** Demographic data of the two groups of participants in the three studies. EFAT: Emotional Face Assessment Task; ERT: Emotion Regulation Task; SEAT: Shifting Emotion Attention Task.

Study	Number of Participants	M/F	Age
EFAT	52	28/24	24.4 ± 1.2
ERT	49	27/22	23.6 ± 1.3
SEAT	49	27/22	23.7 ± 1.3

**Table 2:** Results of regression analysis: only results with significant gender, or gender by childhood income-to-need ratio effects are presented. EFAT = Emotional Face Assessment Task, ERT: Emotion Regulation Task, SEAT: Shifted-Attention Emotion Appraisal Task, CITN: childhood income-to-need ratio, AITN: current adult income-to-need ratio.

Task/Brain Region	Beta Coefficient	T-Ratio	Significance Level
EFAT Right Amygdala Fearful > Happy			
Age	−0.43	−1.64	0.11
CITN	−0.78	−2.86	0.007
AITN	0.45	0.126	0.9
Gender	−5.43	−1.75	0.087
Gender by Age	4.73	1.62	0.11
Gender by AITN	−0.28	−0.67	0.51
Gender by CITN	0.80	2.25	0.03
EFAT Right Amygdala Fearful			
Age	−0.61	−2.39	0.02
CITN	−0.65	−2.42	0.02
AITN	0.16	0.05	0.96
Gender	−10.80	−3.58	0.001
Gender by Age	10.17	3.60	0.001
Gender by AITN	0.86	2.48	0.017
Gender by CITN	−0.14	−0.34	0.74
ERT DLPFC Reappraise > Maintain			
Age	−0.63	−2.12	0.04
CITN	−5.41	−1.20	0.05
AITN	4.16	1.03	0.3
Gender	−5.01	−2.24	0.03
Gender by Age	4.98	2.23	0.03
Gender by AITN	−4.17	−1.04	0.30
Gender by CITN	5.71	2.18	0.03
ERT VLPFC Reappraise > Maintain			
Age	−0.53	−1.58	0.12
CITN	−5.67	−1.84	0.07
AITN	6.73	1.47	0.15
Gender	−5.20	−2.05	0.046
Gender by Age	5.21	2.07	0.05
Gender by AITN	−6.82	−1.51	0.14
Gender by CITN	5.78	1.95	0.05

## Abbreviations

AAL	Automated Anatomical Labeling
DLPFC	Dorsolateral Prefrontal Cortex
DSM	Diagnostic and Statistical Manual
EFAT	Emotional Face Assessment Task
ERT	Emotion Regulation Task
MRI	Magnetic Resonance Imaging
SEAT	Shifting Emotion Attention Task
VLPFC	ventrolateral Prefrontal Cortex
